# Proteins Inside the HSP60/HSP10 Fold Under a Constant Electric Field: Potential Implications for the Protein Folding Problem

**DOI:** 10.3390/ijms27073297

**Published:** 2026-04-05

**Authors:** Lucía J. Peña-Ortiz, Julio Manuel Hernández-Pérez, Bertha Alicia León-Chávez, Jose R. Eguibar, Juan Manuel Solano-Altamirano, Viridiana Vargas-Castro

**Affiliations:** 1 Posgrado en Ciencias Químicas, Benemérita Universidad Autónoma de Puebla, 14 Sur y Av. San Claudio, Col. San Manuel, Puebla C. P. 72570, Puebla, Mexico; lucia.penaort@alumno.buap.mx; 2 Facultad de Ciencias Químicas, Benemérita Unversidad Autónoma de Puebla, 14 Sur y Av. San Claudio, Col. San Manuel, Puebla C. P. 72570, Puebla, Mexico; julio.hernandez@correo.buap.mx (J.M.H.-P.); bertha.leon@correo.buap.mx (B.A.L.-C.); 3 Instituto de Fisiología, Benemérita Unversidad Autónoma de Puebla, 14 Sur #6301, Col. San Manuel, Puebla C. P. 72570, Puebla, Mexico; jose.eguibar@correo.buap.mx

**Keywords:** chaperonin HSP60-HSP10, effective force field, protein folding, foldon, Rhodanese, molecular dynamics simulation

## Abstract

For a protein to perform its biological functions, it must adopt a specific three-dimensional conformation. In addition, many proteins require the assistance of other protein complexes known as chaperonins to fold —i.e., to acquire such a specific conformation—, although the exact mechanisms whereby the chaperonins act and assist the folding process have not been completely determined. In this work, we characterize the physical environment at the interior of the chaperonin HSP60/HSP10 via Molecular Dynamics Simulations. We found that, inside the cavity of the chaperonin (within a region covering much of the cavity’s volume), the long-range electrostatic potential presents a structured pattern that, except for small fluctuations, does not change in time. The electrostatic potential generates an electric field that can be modeled, as a first approximation, as constant and unidirectional $(E/\left(V\;\cdot \;{Å}^{-1}\right)\approx -0.0054\hat{𝚤}+0.010\hat{𝚥}-0.162\hat{k}$, here the chaperonin’s main axis is aligned along $\hat{k})$, which can produce large deformations in the structure of a heated protein (Rhodanese); the long-range approximated E(r) can in fact unfold the Rhodanese, when applied as an external field. Finally, we discuss the possible implications of such an electric field for the protein folding problem, within the context of proteins whose folding is assisted by chaperones. The existence and effects of the electric field are consistent with several theories and experimental observations related to the protein folding problem, in particular with the foldon view.

## 1. Introduction

Proteins are polymers of amino acids that bind together through peptidic bonds. These macromolecules range from a couple of tens of amino acids to enormous complexes of thousands of amino acids. The large number of amino acids entails an astronomical number of possible stable conformations. However, to perform its biological functions, each protein is folded into (i.e., acquires) a specific three-dimensional structure called native structure. In the early 1970’s, Anfinsen proposed and popularized the idea, known as the *Anfinsen’s thermodynamic hypothesis* or simply *thermodynamic view*, that the Gibbs energy of the native structure must be a global minimum of the Gibbs energy landscape (GEL), out of all possible conformations compatible with the physiological environment wherein the protein lives  [1], and that this 3D structure is somehow solely and intrinsically determined by the aminoacidic sequence. The question of why and how the amino acid sequence alone determines the native structure is known as the protein folding problem (PFP). Naturally, the PFP comprises many subproblems that need to be addressed individually or collectively.

Despite many impressive advances in technology, computational techniques, and computing power, which have led to the acquisition of vast amounts of theoretical, computational, and experimental information about the mechanisms by which living organisms fold proteins, the PFP remains far from solved. With the release of AlphaFold [2] and RoseTTAFold  [3], whose developers were later awarded the 2024 Nobel prize, we have gained access to knowing the protein folds, but not the protein folding (as Chen et al. cleverly stated [4]). That is, for a given amino acid sequence, if it is sufficiently similar to sequences whose native structures are known, then the sequence’s native structure can be predicted with fair confidence. Whereas knowing the protein folds is a huge breakthrough, we still have not completely understood in physical and chemical (and even mathematical) terms why the sequence adopts the native structure  [4,5,6,7,8,9].

As in many physical and chemical phenomena, the PFP involves both thermodynamic and kinetic aspects, which are often studied separately. Under this light, several models have been proposed to address the PFP. Among them, we found the hydrophobic collapse model [10,11], foldon or the defined pathway model [12,13], backbone-based folding theory [4,14,15], nucleation condensation model [16], and surely many others that escape our knowledge. Notably, the energy landscape theory [17,18,19,20] constitutes a framework in which concepts such as the funnel-shaped landscapes have arisen, which, in turn, allow for the explanation of many experimental observations [18].

Amid the many challenges that remain unsolved, it seems that there is no consensus about the path or paths a protein follows from the amino-acid-by-amino-acid binding in the ribosome up to the final native structure. In fact, for a long time, it was believed that proteins fold through predetermined pathways [12,21,22]. More recently, Englander et. al proposed that proteins are composed of foldons —building blocks that fold/unfold as units  [12,13,23,24,25], and that the foldon-foldon interactions must, at least, play an important role in determining the folding pathway [12,13].

In this context, two main protein categories can be recognized: proteins that reach their native state if left alone, and proteins that require the assistance of other proteins called chaperones to finalize their folding [4]. Hereafter, we will use the term folding-assisted to refer to the latter proteins. It follows naturally that the folding pathway and mechanism differ for each category, although both types are subjected to the same physical principles, i.e., the folded protein should correspond to a local or global minimum of the GEL. Most likely, the native protein is dynamically moving between different close local GEL minima [26]. In this work, we will focus on folding-assisted mechanism, since the folding of proteins not assisted by chaperones (small fast-folding proteins) can be understood in terms of the funnel-shaped landscape theory.

Other major challenges of the PFP are the facts that proteins reach the native structure in a very rapid range of times, from a few nanoseconds to a few seconds [7,27], and that the folding processes involve timescales from picoseconds to seconds [11]. This is related to the famous Levinthal’s paradox [21,22], which can be simplified as follows. If the amino acid sequence were to explore the entire GEL, it would take longer than the universe’s age to reach the native state due to its huge number of degrees of freedom. Levinthal’s paradox has been solved for small, fast-folding proteins through the energy landscape theory (see, e.g., Ref. [18] and references therein). However, the paradox has not been completely solved for more complex and larger proteins, in particular for a class that requires the assistance of other proteins to reach their native state. It has been experimentally shown that chaperones (and the subfamily called chaperonins) assist other proteins in avoiding transit throughout the entire GEL. A clear example is the eukaryotic chaperonin TRiC/CCT, as revealed by studies on its essential client β-tubulin [28]. Gestaut et al. found that, according to the observations, TRiC assists the β-tubulin to achieve the native structure by providing not only a favorable environment, but also providing guidance through sequential instructions.

Another example is the HSP60/10 chaperonin family, which is present in most life forms [29]. This family is a barrel-shaped complex that has an internal negatively charged cavity [30]. The amount of negative charges varies from species to species, and it is believed that these variations might be the evolutionary response to mutations of the chaperonin’s substrates [30]. However, in this work, we are interested in the possible contribution that chaperonins of this type may have on the general aspects of the PFP. In this context, it has been recognized that the negatively charged cavity wall accelerates the folding process via repulsions between the substrate and the wall [31], and Sadat et al. showed that the cavity acts by lowering the entropy barriers [30]. However, the mechanism whereby the chaperonins accelerate the folding process has not been elucidated.

In this work, we analyze through molecular dynamics simulations the physical environment within the cavity of the human mitochondrial chaperonin  [32], which is known to interact with ∼160 proteins [33], and which we take as a representative of the HSP60/10 family. We aim to extract physical information that may help understand how folding-assisted proteins can reach their native state in short times. We will consider that folding-assisted proteins are transported in pre-folded states, i.e., that the protein does not fold from a fully extended polypeptide chain, but rather reaches the chaperonin in a pre-folded state to complete the last stage of the folding process. Although this might not be a universal mechanism for all folding-assisted proteins, it is known to occur for proteins that interact with the prefoldin [34,35]. This is particularly important when accepting that a protein is composed of foldons, since a natural question arises: how are entire foldons moved as units to reach the native state? Obviously, small isolated interatomic forces do not suffice to cause mesoscopic movements, but larger forces resulting from collective contributions are required.

The organization of this article is as follows. In Section 2.1 we provide the details of the MD simulations of the HSP60/HSP10. In Section 2.2, we describe the details of how, from the MD simulation data, we computed the long-range electrostatic potential, under the Ewald particle-mesh decomposition, throughout the chaperonin and how we averaged it over time (we will denote this time-averaged property as $\langle \phi_{lr}\left(r\right)\rangle$). Subsequently, in Section 2.3, we provide the details of how we extracted the values of $\langle \phi_{lr}\left(r\right)\rangle$ located at the interior of one of the chaperonin’s cavities: briefly, the extraction was performed by determining an ellipsoid that fits inside the upper cavity, and that does not touch the interior atoms of the chaperonin. To extract useful information, we kept only the values of $\langle \phi_{lr}\left(r\right)\rangle$ that are located inside the ellipsoid. The data was further filtered so that values of $\langle \phi_{lr}\left(r\right)\rangle$ with high fluctuations were discarded. In Section 2.4 we describe how we determined a mathematical expression for $\langle \phi_{lr}\left(r\right)\rangle$: The filtered data, which we call control points, were fitted to polynomials of *x*, *y* and *z* using the least squares method. We tested two approximations, one linear in the coordinates and other with polynomials of order 2 in the coordinates. From the polynomial fits, we computed the electric field by taking the negative gradient of $\langle \phi_{lr}\left(r\right)\rangle$. The expressions so obtained represent the long-range electric field inside the upper chaperonin cavity, away from the atoms of the chaperonine. The approximations are valid rigorously only within the ellipsoid, i.e., only within a part of the complete upper chaperonin cavity. From the two polynomial expansions, the simplest form ($\langle \phi_{lr}\left(r\right)\rangle$ linear in *x*, *y*, and *z*) renders a constant long-range electric field E(r). Further analysis shows that the $E_{z}$ component is the largest component regardless the order of the polynomial expansion. Thus, in its simplest form, the electric field inside the approximated cavity (i.e., the ellipsoid that covers part of the upper chaperonin cavity) can be further approximated as a constant electric field along the main chaperonin axis. In Section 2.4.3, as a proof-of-concept, we test the effect of the most simple form of the electric field applied to a heated protein (Rhodanese). The applied electric field was tested alone and extrapolated over a 3D space larger than the size of the ellipsoid (i.e., over the space required to study the MD simulation of the Rhodanese). In this context, the studied effect must not be confused with the complete effect of the chaperonin over a protein, since the test does not consider the effects of the confinement nor the negative-charged nature of the chaperonin. However, the test provides details of what the electric field alone may do on the Rhodanese. We found that the electric field induces large deformations in the protein, causing its unfolding. In Section 3 we discuss the possible implications of the existence of a resultant long-range electric field that is present over the selected region studied in this article on the protein folding problem, specifically for the chaperone-assisted proteins. In Section 4 we provide further technical details of simulations, finding the ellipsoid location (inside the upper chaperonin cavity) and its size, and the specific least-square method details applied in the article. Finally, in Section 5 we provide some conclusions.

## 2. Results

### 2.1. Molecular Dynamics of HSP60/HSP10

**Figure 1:**
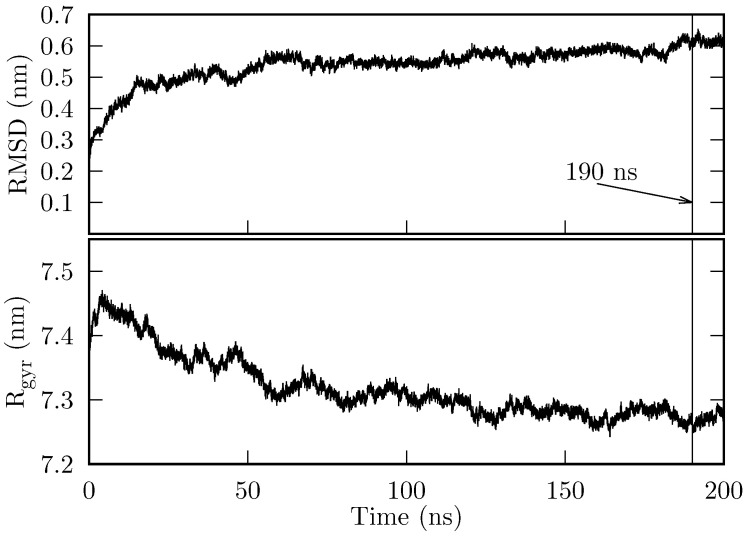
RMSD and $R_{\mathrm{gyr}}$ of the HSP60/HSP10 production MD. Both RMSD and $R_{\mathrm{gyr}}$ are computed considering all atoms.

The initial state of the chaperonin HSP60/HSP10 (hereafter, we will refer to this system simply as the chaperonin) was prepared as described in Section 4.1. 200 ns of production simulation were obtained. In Figure 1, we show the root mean square deviation (RMSD) and the radius of gyration ($R_{\mathrm{gyr}}$). The chaperonin’s RMSD reaches a stable value of ∼0.55 nm at around 140 ns, and jumps to ∼0.6 nm at approximately 185 ns. During the final 10 ns, the system’s RMSD remains at 0.6 nm. On the other hand, $R_{\mathrm{gyr}}$ converges to (∼$7.25\leq R_{g}\leq 7.3$ nm), from approximately 120 ns onwards.

Both the RMDS and $R_{\mathrm{gyr}}$ show that the system is stable over time, i.e., it does not suffer denaturation. In particular, the last 10 ns are sufficiently stable for our purposes.

### 2.2. The Long-Range Electrostatic Potential Inside the Chaperonin

From the MD simulation trajectory, we extracted 1000 frames corresponding to the last 10 ns of the production simulation. Using the atoms of the first frame, we determined the dimensions of a box that encloses all chaperonin atoms, and centered this box at the chaperonin’s mass center. The long-range electrostatic potential, $\phi_{lr}$, was computed using the VMD’s plugin PMEPot on a grid that covers the previous box. For each subsequent frame, we aligned the chaperonin atoms to the coordinates of the first frame, and for computing $\phi_{lr}$, we used the same box dimensions, but we centered the box at the chaperonin center of mass (of the respective frame). Assuming that the grid contains superposable information, we computed the average and standard deviation at each grid point over all frames. We found that $\langle \phi_{lr}\left(r_{i}\right)\rangle$ (here $r_{i}$ is the coordinate of the *i*-th grid point) varies on the grid from −13.87 V to 11.47 V, and $\sigma (r_{i})$ varies from 0.04 V to 1.96 V. The largest values of $\langle \phi_{lr}\left(r_{i}\right)\rangle$ and σ are located around the zones where atoms are present.

**Figure 2:**
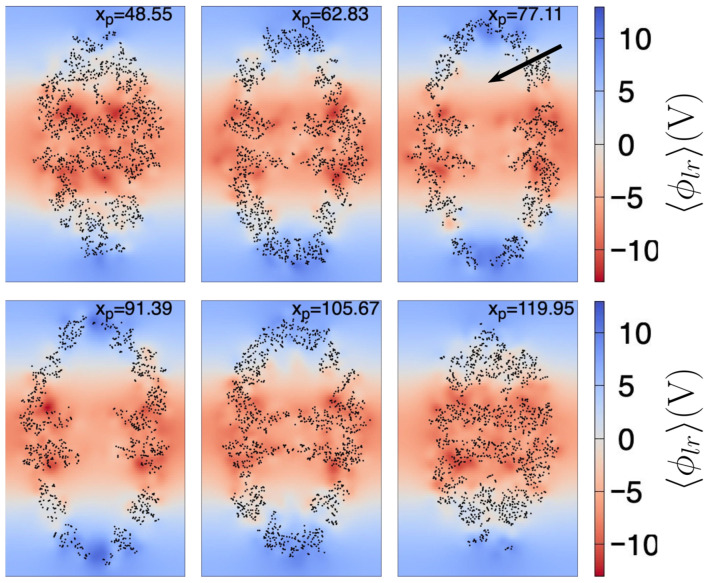
Mean long-range electrostatic potential, $\langle \phi_{lr}\rangle$, throughout the chaperonin. Each subfigure (171.3 Å width by 263.1 Å height) shows the $\langle \phi_{lr}\rangle$ on an intersecting yz plane, located at the $x_{p}$ position (in Å) depicted in the top of the subfigure. Black dots are chaperonin atoms with $|x_{\alpha }-x_{p}|\;\leq \;0.5$ Å, where $x_{\alpha }$ is the coordinate of the atom α (of the first frame). The color scale indicated at the right side is the same for all subfigures. The black arrow shown in the right top subfigure points the approximate center of the upper cavity.

In Figure 2, we show $\langle \phi_{lr}\left(r\right)\rangle$ at different yz planes, each of which cuts the chaperonin at the $x_{p}$ value shown at the top of each plane. Here, the largest dimension of the chaperonin is aligned to the *z* axis. We can observe that $\langle \phi_{lr}\left(r\right)\rangle$ from the center to the top of the chaperonin increases in value, especially at the chaperonin cavity (the approximate center of the upper cavity is indicated by the black arrow in Figure 2). Not surprisingly, near the atoms $\langle \phi_{lr}\left(r\right)\rangle$ is larger, relative to the zones without atoms. However, in this work we are mainly interested in the shape of $\langle \phi_{lr}\rangle$ throughout the chaperonin cavity, thus we can observe (see planes with $x_{p}=62.83,\;77.11,\;91.39,105.67$) that $\langle \phi_{lr}\rangle$ shows a negative-to-positive trend around the center of the upper chaperonin cavity (the reverse trend is observed in the lower cavity).

**Figure 3:**
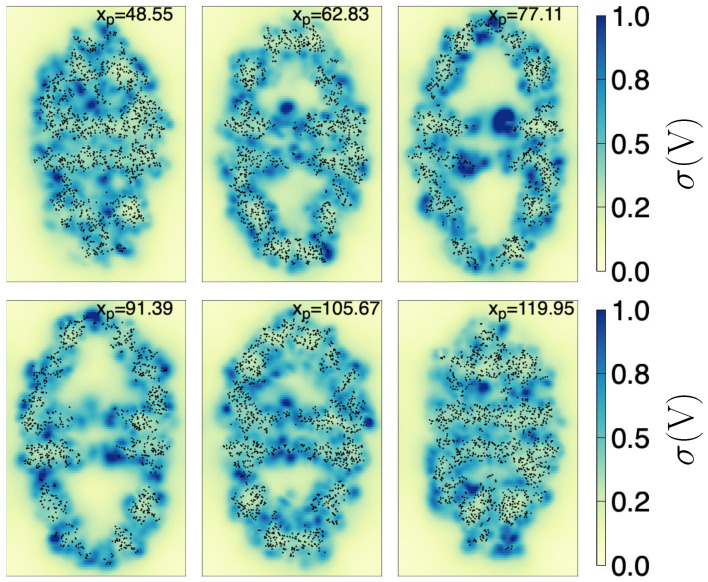
Standard deviation, σ, of the long-range electrostatic potential, throughout the chaperonin. Each subfigure (171.3 Å width by 263.1 Å height) shows the σ on an intersecting yz plane, located at the *x* position (in Å) depicted in the top of the subfigure. Black dots are chaperonin atoms with $|x_{\alpha }-x_{p}|\;\leq \;0.5$ Å, where $x_{\alpha }$ is the coordinate of the atom α (of the first frame). The color scale indicated at the right side is the same for all subfigures.

Furthermore, in Figure 3, we show the standard deviation of $\langle \phi_{lr}\rangle$ (σ) throughout the chaperonin. The planes cut the chaperonin at the same locations as in Figure 2. We see that around the center of both cavities (upper and lower), σ is generally close to zero. This implies not only that $\langle \phi_{lr}\left(r\right)\rangle$ have a definite structure (from negative to positive along the *z*-axis inside the upper cavity —and viceversa in the lower cavity) but also that it can be considered almost invariant in time. As additional information, the zone near the center of the chaperonin (lowest part of the upper cavity) fluctuates the most, possibly because the nearby atoms are moving the most (see the bluest zone on the plane with $x_{p}=77.11$).

So far, we have been focusing on analyzing the upper cavity region. This is so because it is well known that the HSP60/HSP10 chaperonin (which is similar to the GroEL/GroES family) forms two confined folding chambers. The folding of a protein occurs only at one of these chambers (i.e., once inside of one cavity, the protein cannot migrate towards the other by crossing the chaperonin interior), and the respective chamber space is mostly occupied by the protein to be folded [31]. Thus, in what follows, unless stated otherwise, we will focus only on the upper cavity region, which we refer to simply as the cavity. The lower cavity properties should be similar to the upper’s, save for a mirror symmetry relative to the xy plane.

### 2.3. Control Points

Since we are interested in characterizing the long-range electrostatic potential within the cavity, we extracted the relevant $\langle \phi_{lr}\left(r_{i}\right)\rangle$, i.e., the $\langle \phi_{lr}\rangle$ evaluated at grid points inside the cavity that contain relevant information. To this end, we used two criteria. First, the grid point must be inside the cavity. Second, the $\langle \phi_{lr}\left(r_{i}\right)\rangle$ must have a small σ.

**Figure 4:**
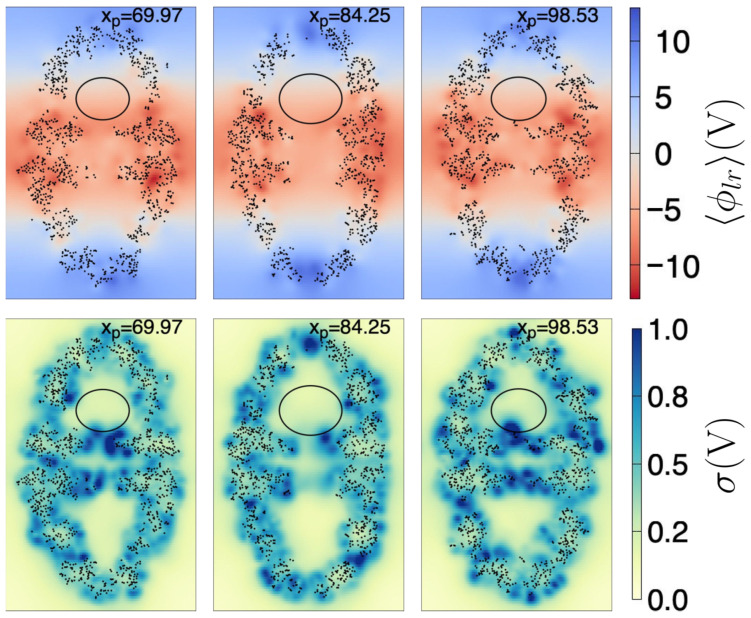
Plane cuts of $\langle \phi_{lr}\left(r\right)\rangle$ (upper row) and σ (lower row). Each yz plane (171.3 Å width by 263.1 Å height) intersects the chaperonin at the $x_{p}$ position (in Å) depicted in the top of the respective subfigure. Black dots are chaperonin atoms with $|x_{\alpha }-x_{p}|\;\leq \;0.5$ Å, where $x_{\alpha }$ is the coordinate of the atom α (of the first frame). The ellipse is the intersection curve of the ellipsoid found as described in the text. The color scale indicated at the right side is the same for all subfigures to its left.

As a first approximation, to ensure a grid point is within the cavity, we determined the largest ellipsoid that fits inside the cavity, i.e., that it does not touch the interior atoms, taking as reference the coordinates of the first frame (see Section 4.3 for further details). The found ellipsoid has radii a=28.11 Å, b=28.16 Å, and c=22.32 Å, and center coordinates $c_{x}=188.875$ Å, $c_{y}=188.341$ Å, and $c_{z}=235.258$ Å. Figure 4 depicts a few plane cuts of the chaperonin and the ellipsoid, both for the $\langle \phi_{lr}\left(r\right)\rangle$ and for σ.

**Figure 5:**
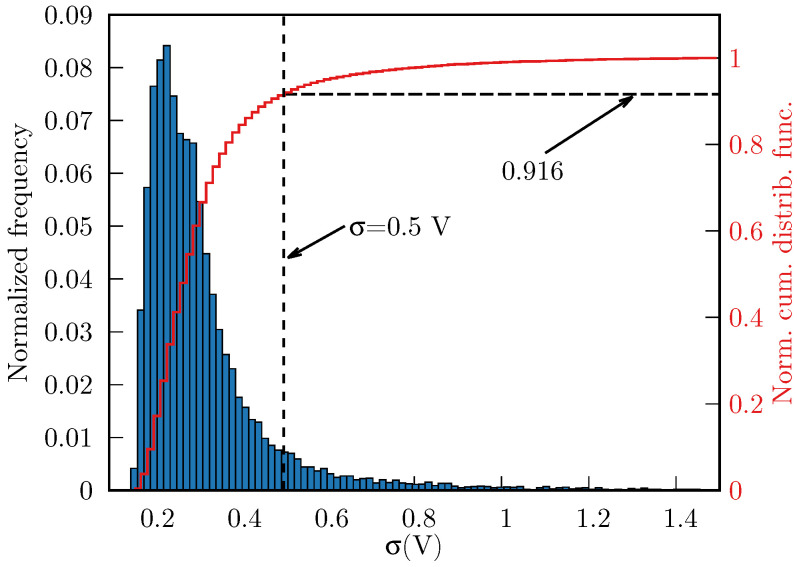
Normalized histogram of $\sigma (r_{i})$ (blue boxes, 16,551 points in total) and the normal cumulative distribution function (red line), inside the ellipsoid. The threshold σ=0.5 V (vertical dashed line) removes ∼8.4% of points with largest fluctuations.

We found 16,551 grid points to be inside the ellipsoid. Subsequently, we discarded the grid points whose σ≤0.5 V, thereby eliminating 8.4% of the points enclosed by the ellipsoid, i.e., we obtained 15,160 points that met both above criteria. In Figure 5, we show the distribution of σ throughout the cavity (normalized frequencies and normal cumulative distribution function).

Removing the points with high σ left us with information that is located far from the atoms of the chaperonin, relative to the first frame we used to compute $\langle \phi_{lr}\left(r\right)\rangle$ and σ (see Section 4.2 for specific details). These fluctuations are most likely caused by highly-movable atoms, that are entering and exiting the ellipsoid at different times. Therefore, this information is not meaningful to our purposes. Furthermore, we used the ellipsoid only as a mean to extract information from the interior of the chaperonin cavity, and in doing so we are missing points that are inside the cavity, but outside the ellipsoid. Nonetheless, the filtered information provides a sampling of the chaperonin’s cavity that will be useful to determine an approximated mathematical expression for $\langle \phi_{lr}\left(r\right)\rangle$ and its corresponding long-range electric field.

### 2.4. Polynomial Fitting

#### 2.4.1. Long-Range Electrostatic Potential

The control points obtained in Section 2.3 were used to obtain an approximate equation of $\langle \phi_{lr}\left(r\right)\rangle$ inside the chaperonin cavity. To this end, we fitted the control points using the least square method (see Section 4.4 for specific details) to the following two equations:

(1) $$\langle \phi_{lr}\left(r\right)\rangle =A_{0}+A_{x}x+A_{y}y+A_{z}z,$$

and

(2) $$\begin{array}{ccc} \langle \phi_{lr}^{\prime }\left(r\right)\rangle & = & A_{0}^{\prime }+A_{x}^{\prime }x+A_{y}^{\prime }y+A_{z}^{\prime }z+\\ & & A_{xx}^{\prime }x^{2}+A_{xy}^{\prime }xy+A_{xz}^{\prime }xz+\\ & & A_{yy}^{\prime }y^{2}+A_{yz}^{\prime }yz+A_{zz}^{\prime }z^{2}. \end{array}$$

In simple terms, we tested linear and quadratic equations in the coordinates. It must be noted that Equations (1) and (2) are valid within the ellipsoid of equation:

(3) $$\frac{{\left(x-c_{x}\right)}^{2}}{a^{2}}+\frac{{\left(y-c_{y}\right)}^{2}}{b^{2}}+\frac{{\left(z-c_{z}\right)}^{2}}{c^{2}}=1,$$

where $c_{x}=188.875$ Å, $c_{y}=188.341$ Å, $c_{z}=235.258$ Å, a=28.11 Å, b=28.16 Å, and c=22.32 Å. The set of control point coordinates found as described in Section 2.3 (i.e., the control points located inside the ellipsoid of Equation (3)), together with the values of $\langle \phi_{lr}\left(r_{i}\right)\rangle$ and $\sigma (r_{i})$ are provided as a Supporting Information file. Specific details of how $\langle \phi_{lr}\left(r_{i}\right)\rangle$ and $\sigma (r_{i})$ were determined can be found in Section 4.2.

After fitting, we found the coefficients shown in Table 1, and to determine the quality of the fit, we calculated the coefficients of determination, as shown in Table 2.

The coefficient of determination for $\langle \phi_{lr}^{\prime }\left(r\right)\rangle$ is greater than for the linear expansion; however, the linear expression (Equation (1)) is easier to test in MD programs such as GROMACS and LAMMPS. We leave it to the interested reader to choose between the two approximations; we provide them here only for completeness and for future reference.

**Figure 6:**
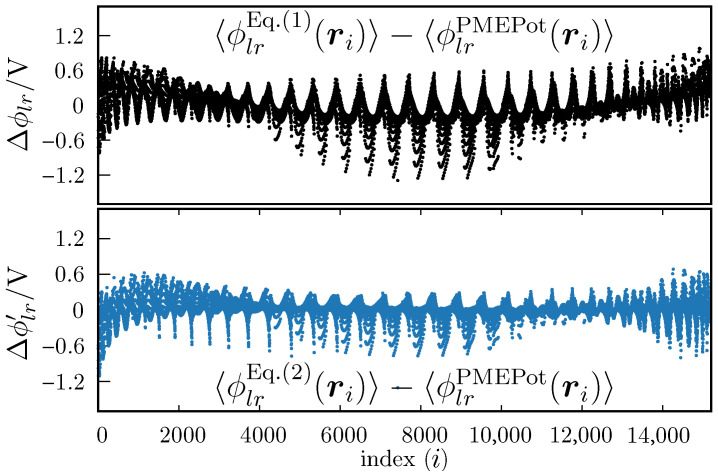
Differences between fitted and PMEPot values of the electrostatic potential, $\langle \phi_{lr}\left(r_{i}\right)\rangle$. The upper plot is for the fit against Equation (1), and the lower plot is for the fit against Equation (2). The abscissa is an index associated with an ordered list of points $r_{i}$. All $r_{i}$ are located within the ellipsoid defined by Equation (3), and the order is not representative of the position in the 3D Cartesian coordinate system.

In Figure 6 we show the differences between the measured $\langle \phi_{lr}\left(r_{i}\right)\rangle$ (i.e., the values of $\langle \phi_{lr}\rangle$ at each control point) vs. the value obtained with Equation (1) (upper plot) or Equation (2) (lower plot). We observe that the differences are slightly greater for Equation (1), which is not surprising. However the differences are not overwhelmingly greater. That is, the linear fit (Equation (1) seems to be a good approximation, relative to the fit against Equation (2). This is also reflected by the fact that the determination coefficients are close to each other.

#### 2.4.2. Long-Range Electrostatic Field

Taking the negative gradient of Equation (1) renders the mean long-range electric field inside the chaperonin’s cavity, which we will denote simply as E(r). i.e., under the simplest approximation, the electric field inside the chaperonin cavity has the constant form:

(4) $$E\left(r\right)=-A_{x}\hat{𝚤}-A_{y}\hat{𝚥}-A_{z}\hat{k}.$$

From Table 1, we see that $A_{x}=0.034A_{z}$ and $A_{y}=0.065A_{z}$, thus E(r) within the cavity can be considered constant and for practical purposes along the main axis of the chaperonin.

As we commented above, Equation (4) constitutes the simplest approximation for E(r) inside the ellipsoidal region contained, in turn, within the chaperonin cavity. However, this simple constant form will allow us to test the long-range electric field effects in MD simulations, as programs such as GROMACS or LAMMPS have already built-in instructions for applying an external electric field, i.e., we can immediately perform MD simulations without modifying the code of these programs. This fact adds to our choice of ignoring for now the slightly better fitting of the squared expansion (Equation (2)).

Nevertheless, for future reference, we also provide the electric field obtained by taking the negative gradient of $\langle \phi_{lr}^{\prime }\left(r\right)\rangle$, i.e., the negative gradient of Equation (2)) is:

(5) $$\begin{array}{ccc} E^{\prime }\left(r\right) & = & -\left(A_{x}^{\prime }+2A_{xx}^{\prime }x+A_{xy}^{\prime }y+A_{xz}^{\prime }z\right)\hat{𝚤}\\ & & -\left(A_{y}^{\prime }+A_{xy}^{\prime }x+2A_{yy}^{\prime }y+A_{yz}^{\prime }z\right)\hat{𝚥}\\ & & -\left(A_{z}^{\prime }+A_{xz}^{\prime }x+A_{yz}^{\prime }y+2A_{zz}^{\prime }z\right)\hat{k}, \end{array}$$

which is also valid for points that satisfy Equation (3), i.e., for points inside the cavity.

**Figure 7:**
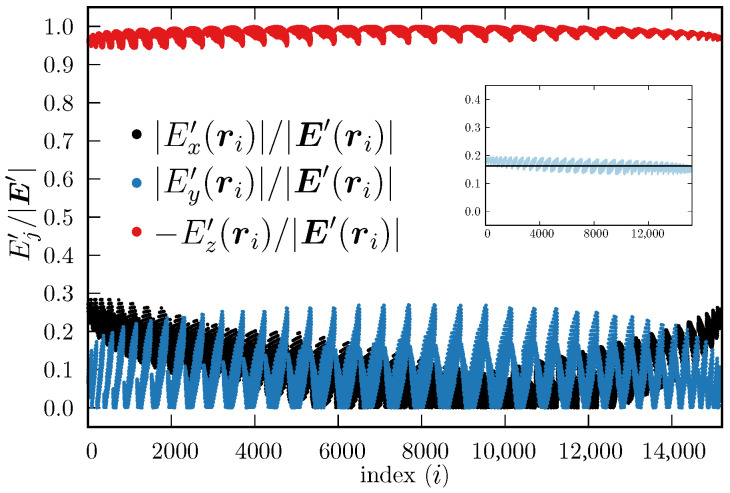
Components of $E^{\prime }$ (Equation (5)), evaluated at the control points, relative to their respective total magnitude. The index *i*, shown in the abscissas, represents the place of a control point $r_{i}$ within an ordered list (all $r_{i}$ are located within the ellipsoid defined by Equation (3)). The order is not representative of the location in the 3D Cartesian system. Black and blue dots represent the absolute value of the relative components $|E_{x}^{\prime }\left(r_{i}\right)|/|E\left(r_{i}\right)|$ and $|E_{y}^{\prime }\left(r_{i}\right)\left|/\right|E^{\prime }\left(r_{i}\right)|$, respectively, whereas the red dots depict $-E_{z}^{\prime }\left(r_{i}\right)/\left|E^{\prime }\left(r_{i}\right)\right|$; here $|E^{\prime }\left(r_{i}\right)|\equiv \sqrt{E^{\prime }\left(r_{i}\right)\;\cdot \;E^{\prime }\left(r_{i}\right)}$. Inset: $\left|E(r_{i})\right|$ (Equation (4), black line, in V·Å^−1^) and $|E^{\prime }\left(r_{i}\right)|$ (Equation (5), light blue dots, in V·Å^−1^).

Figure 7 shows the components of the electric field calculated using Equation (5) evaluated at each control point $r_{i}$, relative to its total magnitude, i.e., $E_{j}^{\prime }\left(r_{i}\right)/\left|E^{\prime }\left(r_{i}\right)\right|$, j=x,y,z. To facilitate the visualization, we plotted the absolute values $|E_{x}^{\prime }\left(r_{i}\right)\left|/\right|E^{\prime }\left(r_{i}\right)|$ (black dots) and $|E_{y}^{\prime }\left(r_{i}\right)\left|/\right|E^{\prime }\left(r_{i}\right)|$ (blue dots), but we plotted $-E_{z}^{\prime }\left(r_{i}\right)/\left|E^{\prime }\left(r_{i}\right)\right|$ (red dots). This helps to observe that the $E_{z}^{\prime }\left(r_{i}\right)$ component does not change sign, whereas $E_{x}^{\prime }\left(r_{i}\right)$ and $E_{y}^{\prime }\left(r_{i}\right)$ may do, depending on the specific values $x_{i}$, $y_{i}$ and $z_{i}$. From Figure 7, it is observed that the magnitude of $E_{z}^{\prime }\left(r_{i}\right)$ is always considerably larger than the other two components, which also supports the proposal that the electric field inside the chaperonin cavity (as sampled by the ellipsoidal region) can be simplified to be a constant electric field along the *z* axis (actually along the main inertia axis of the chaperonin). Furthermore, the magnitudes of the electric field obtained with Equation (4) and with Equation (5) have similar magnitudes, as can be seen in the inset of Figure 7, which shows that Equation (4) captures most of the electric field essence inside the chaperonin cavity. In addition, the fact that the $E_{z}$ component is the largest in magnitude out of the three, supports the proposal that the electric field can be further simplified to be $E\approx \left(-0.162\;V\;\cdot \;{Å}^{-1}\right)\hat{k}$, which is an expression that can be used already in MD simulations. We emphasize again that every control point $r_{i}$ is located within the approximated upper cavity of the chaperonin, i.e., within the ellipsoid defined by Equation (3); hence, Equations (4) and (5) are valid throughout the ellipsoid.

**Figure 8:**
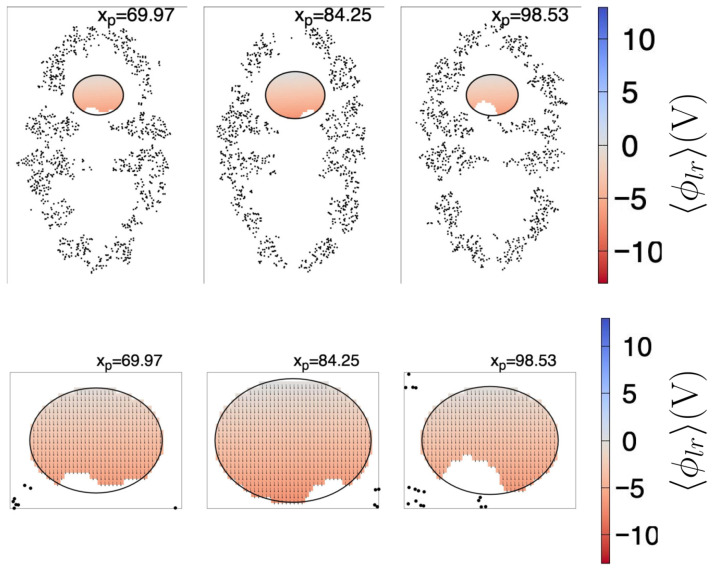
Plane cuts of the fitted $\langle \phi_{lr}\left(r\right)\rangle$ (Equation (1), upper row) and projected E(r) (Equation (4), lower row). For visualization purposes, the size of the arrows in the lower plot are scaled by a factor of 10, i.e., the arrows are $10(-A_{y}\hat{𝚥}-A_{z}\hat{k})$; the $-A_{x}\hat{𝚤}$ component exits the shown plane, thus it was not draw, and the plot sizes have been magnified. Each upper row yz plane (171.3 Å width by 263.1 Å height) intersects the chaperonin at the $x_{p}$ position (in Å) depicted in the top of the respective subfigure. Black dots are chaperonin atoms with $|x_{\alpha }-x_{p}|\;\leq \;0.5$ Å, where $x_{\alpha }$ is the coordinate of the atom α (of the first frame). The ellipse is the intersection curve of the ellipsoid found as described in the text. The color scale indicated at the right side is the same for all subfigures to its left. Only the domains wherein Equations (1) and (4) are valid are colored, and missing points inside the ellipsoids correspond to control points with σ>0.5 V.

In Figure 8, we show the fitted $\langle \phi_{lr}\left(r\right)\rangle$ (Equation (1)) together with the resultant projected E(r), for the same plane cuts shown in Figure 4. For visualization purposes, the size of the vectors depicted in the lower row are $10(-A_{y}\hat{𝚥}-A_{z}\hat{k})$; the $E_{x}\hat{𝚤}$ exits the plane, thus the component was not drawn. The colored regions correspond to the cuts to the ellipsoid given by Equation (3), wherein Equations (1)–(5) are valid. The missing points inside the ellipsoid correspond to the coordinates of the control points with $\sigma (r_{i})<0.5$ V. As can be seen, the electric field is predominantly aligned to the *z* coordinate, even if $E_{y}\neq 0$.

From a certain point of view, the fitted Equations (1)–(5) are valid only at the control points, i.e., in the white regions of the ellipsoid (as shown in Figure 8), and some readers may consider the approximated Equations (1)–(5) do not apply. However, for modeling purposes, the resultant expressions can be considered approximately valid even outside the ellipsoid, and valid within the interior of the upper cavity (obviously sufficiently far from the chaperonin atoms). Let us recall that the ellipsoid is a geometric artifact, useful to extract information from the cavity, but it must not be confused with the cavity itself. Furthermore, once we have extracted the approximated mathematical expression of the long-range electric field, and particularly once we have shown that $E_{z}$ is the largest component in magnitude, we can use the simplest form of the long-range electric field ($E\left(r\right)\approx -A_{z}\hat{k}$) in MD simulations as a first approximation.

We close this section by noticing that the electrostatic field inside the lower cavity shows a similar mirrored (relative to the *z* axis) pattern as the upper cavity. Although we did not characterize the lower cavity, intuitively, a very similar long-range resultant electric field should be present.

#### 2.4.3. Proof of Concept

In this section, we apply the electric field found in the previous section to a heated Rhodanese protein (Rhodanese PDB-ID: 8AGF). We apply the long-range electric field as an external field, without the chaperonin and without considering the confining effects. For this, we used the same procedure and configuration parameters as described in Section 4.1. We set up the protein within an 8.34067 × 8.34067 × 8.34067 (nm^3^) cubic box and added 17,551 water molecules. We minimized the system for 50,000 steps, then performed an NVT equilibration for 100 ps at 300 K, followed by an NPT equilibration of 100 ps at 300 K and 1 bar, and finally a production MD of 200 ns. Afterward, we heated the protein: 100 ps of NVT equilibration at 318 K, 100 ps of NPT at 318 K and 1 bar, and 2.1 ns production MD at 318 K. This was followed by a cooling from 318 K to 300 K (1.5 ns), and a final equilibration at 300 K for 2 ns. Finally, we performed two 500 ns production simulations with and without a static electric field ($E_{z}=-0.16188$ V/Å). To apply the electric field, in the GROMACS input script, we set up the keyword “electric-field-z = −1.619 0 0 0”; this corresponds to applying a constant electric field $E=\left(-1.619\;V\;\cdot \;{\mathrm{nm}}^{-1}\right)\hat{k}$ throughout the space occupied by the Rhodanese. This system is conceived as a simulation of how the Rhodanese would evolve in time under the influence of the approximated long-range electric field that would be present if the Rhodanese were inside the chaperonin upper cavity, but without including the chaperonin *per se*. We emphasize that this is not to be considered a simulation of the full effects the chaperonin exerts over a protein, but only the effect of the isolated external constant electric field.

**Figure 9:**
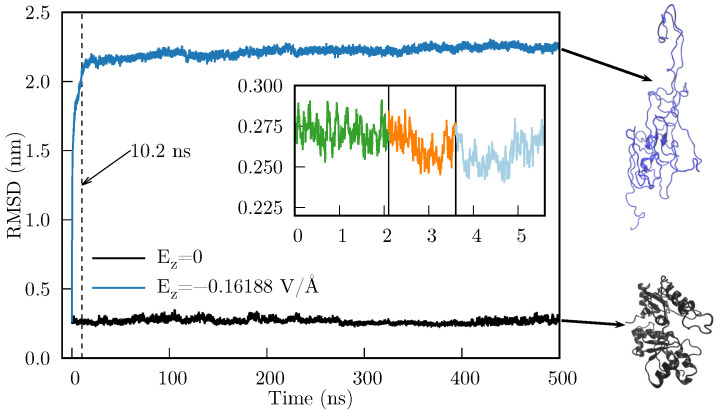
RMSD for the MD simulation of the Rhodanese protein with $E_{z}=0$ (black line) and with $E_{z}=-A_{z}$ (blue line). Inset: heating-cooling simulation which consisted of 2.1 ns of MD simulation at 318 K (green), followed by 1.5 ns of cooling to 300 K (orange), and a final MD equilibration of 2 ns (light blue). Final states of both MD simulations are shown in the right side (generated with VMD [36]).

In Figure 9 we compare the RMSD of the time evolution of the heated Rhodanese with and without an applied $E=E_{z}\hat{k}=-A_{z}\hat{k}$. As we can observe, there is a considerable difference in the RMSD, which indicates that the Rhodanese behaves quite differently under an applied electric field. This implies that the resultant force caused by E suffices to produce mesoscopic changes in the protein. Furthermore, the abrupt change in RMSD occurs during the first 10.2 ns (the dashed line in Figure 9 is located at 10.2 ns).

It should be noted that heating is unlikely to be important for the deformation to occur; the objective of this section is to show that applying an external electric field can largely deform a protein. In fact, since the tested protein is not confined by a wall (as when it is inside the chaperonin), thereby limiting the space in which it can deform, we obtained an unfolded state of the Rhodanese. This unfolding, however, is consistent with the known fact that chaperones need to unfold proteins to refold them to their native state. We are currently conducting studies to analyze whether there is a difference in applying the electric field to a heated vs. non-heated protein. However, this analysis is out of scope for this article, because the folding of a protein likely requires the inclusion of the confining wall.

## 3. Discussion

As Ben-Naim discussed, there are two main approaches to address the PFP: (a) target-based approach, e.g., the folding has the target of reaching a minimum of GEL. (b) cause-based approach, e.g., the folding is caused by certain forces that accelerate and direct the folding process [37]. Perhaps both approaches are not mutually exclusive; maybe we only need the missing link between both approaches, and a better classification of the folding processes. i.e., the influence of the chaperonins may be partially included, especially in MD simulations, as an external electric field. Partially, because the confinement generated by the chaperonin cavity surely has an effect as well. However, the external electric field alone may have identifiable consequences, such as temporarily alter some local GEL barriers (if applied only at certain stages of the folding process), thereby accelerating the passing through local minima. In the above context, attempting to cover all proteins within a single unified approach (by applying fields, external or local, fixed throughout the entire folding period) may constitute an underlying cause of many unsuccessful attempts to achieve a complete understanding of the PFP.

In this work, we focused on folding processes assisted by chaperones, which involve proteins whose folding is more likely to be ruled by foldon-foldon interactions. Our results show that within an important region inside the HSP60/HSP10 chaperonin cavity, and as the simplest approximation, a long-range unidirectional electric field exists, which does not vary significantly over time. Due to the lack of knowledge of measured prefolded states (i.e., we could not obtain an experimentally measured prefolded protein for testing the field found here), we applied the electric field alone to a heated protein. Here, we applied the approximated electric field without the chaperonin, with the purpose of testing exclusivey the electric field effects (obviously, this is not a model of the complete chaperonin). Whereas this might not be representative of the initial folding or refolding processes, it allowed us to confirm that the electric field can cause large deformations, ultimately unfolding the protein, even if the heating process did not denatured the test protein. In the general folding-assisted context, an external field of this kind may contribute to the movement of foldon-sized structural elements until the protein reaches its native structure. Here, it is important to remark that for a better understanding of the PFP, we also need to improve our knowledge on the description of the unfolded protein before it folds, as has been noted for small proteins [38,39,40] and specially in folding-assisted proteins, wherein it is mandatory to know a realistic conformation of the protein when it enters the chaperonin cavity.

The fact that the negative charges of the chaperonin cavity render a resultant external force is also compatible with the idea that solely physical and chemical forces govern the folding process. From this perspective, in both assisted and non-assisted folding, the idea that the folding is driven by explicit forces exerted on the atoms of the amino acid chain [41] and expressed in terms of fundamental interactions makes sense. If we want to perform simulations aimed to understand the folding of a protein within the chaperonin cavity, we may replace the chaperonin long-range electric field with an external electric field. Without this field, the deformation observed in Figure 9 would not occur easily as a consequence of the random atom movements caused by the MD force field and the ensemble algorithm (see the evolution of the protein without the electric field —black line of Figure 9); perhaps the deformation would not occur at all. In this sense, the net force caused by E(r) can move entire portions of the protein that the MD force field alone (without specialized modifications) cannot. These portions can be associated with the foldons or other recent similar concepts, such as the Confined Lowest Energy Fragments proposed by Cao [6] or the building blocks suggested by Škrbić et al. [9].

Another aspect we will test in future work is the inclusion of a confining wall that encloses the unfolded (and preferably pre-folded) protein. Certainly, it has been shown that the confinement provided by the chaperonin cavity is crucial for the correct folding [42]. It has also been proposed that the wall modifies the hydrophobic forces between the unfolded protein and the water inside the chaperonin cavity [43,44]. The results presented in Section 2.4.3 can be considered a first approximation, in the sense that for a better simulation of the last stage of the folding process of a folding-assisted protein, the simulations must include both the electric field found here as well as a confining wall. It remains to be tested whether the confinement alone, or the combination of confinement and the electric field, is the underlying cause of the hydrophobic force modification proposed by England et al.. Also in this context, a common approach to study folding processes consists of modifying the parameters of atomistic force fields, see e.g., [45]. Other approaches are based on the design or specialization of force fields to work specifically with certain proteins or their specific folding, see e.g., [46]. Even more, some approaches propose the existence of external fields that prevent the unfolded protein from acquiring micelle-like structures [47]. It remains to be confirmed that the inclusion of the external electric field together with the confining wall constitutes a better approach to simulate the folding process, as opposed to modifying the parameters of the atomistic force fields. However, the force field alone may still have a real impact on the quality of the simulation of folding of small fast-folding proteins, i.e., non-assisted-folding proteins [48].

In addition, the existence of an external force acting on the unfolded protein is also compatible with the idea that the protein never samples all the GEL [49]. This, in turn, is perfectly consistent with the short times a protein takes to reach its native structure [7,27]. It is also a bridge between the two approaches commented by Ben-Naim [37]: the protein targets a minimum (local or global) of the GEL through a pathway driven by forces (at some point an external electric field) that accelerate and direct the process.

We close this section with a remark. In addressing the yet unsolved protein folding problem, one needs to consider not only general aspects of the process, such as the atomistic forces, the thermodynamic guide to reach a minimum of the Gibbs energy landscape, and so on, but also the specific features of the protein under observation. That is, we need to separate the problem of how small proteins fold by themselves from how proteins that require the assistance of chaperones fold. It is highly likely that in the folding-assisted proteins, the existence of forces strong enough to move foldons is required during the last stage of folding. Under the simplest approximation and throughout an important region, HSP60/HSP10 chaperonin cavities provide an isolated environment, comprised by at least a net, unidirectional, and constant long-range electric field, together with a confining wall in which the last stage of folding occurs.

## 4. Materials and Methods

### 4.1. MD Simulation Details

All HSP60/HSP10 MD simulations were performed using the GROMACS (v. 2023.4) package. The protein geometry was downloaded from the RSCB Protein Data Bank [50] (entry 6MRC), and the OPLS-AA force field [51] was used. The protein was solvated with 436,893 water molecules, and solvent molecules were described by the SPC/E model [52]. 98 sodium counterions were added to achieve electrostatic neutrality. The solvated protein was placed in the center of a 27.436 nm × 27.436 nm × 27.436 nm rhombic dodecahedron box. A 100,000-step energy minimization was performed using the steepest descent method. After energy minimization, two rounds of 100 ps equilibration were performed: first, under the NVT ensemble (310 K) using the modified Berendsen thermostat, and subsequently under the NPT ensemble using the Nose-Hoover Langevin piston (1 bar). The cutoff method was used for van der Waals interactions with a radius of 1.0 nm. The Particle Mesh Ewald (PME) method was used to include long-range electrostatic interactions, with a 1.0 nm cutoff radius for the short-range interactions. Neighbor lists were updated every 500 steps. The LINCS method was used to constrain all bonds. 200 ns of production simulation were performed. NVT and NPT equilibrations, as well as production, were carried out with 2 fs timesteps.

### 4.2. Long-Range Electrostatic Potential

The long-range electrostatic potential at the interior of the chaperonin HSP60-HSP10 (hereafter called simply as the chaperonin) at time $t_{n}$ ($\phi_{lr}\left(t_{n}\right)$) was calculated through the PMEPot plugin (v. 1.0) implemented in VMD [53] (v. 1.9.3). Technical details are provided by Aksimentiev and Schulten [54]. Here, it is important to remark that the PMEPot plugin computes only the long-range potential (i.e., the reciprocal term of the PME decomposition), and that it takes input generated by the program pdb2pqr (v. 3.6.2) [55,56].

The mean long-range electrostatic potential $\langle \phi_{lr}\rangle$ was calculated over N=1000 frames, corresponding to the last 10 ns of the production MD trajectory, keeping only the chaperonin atoms (i.e., water and ions were removed). For each frame, charges and radii for the PME potential were generated using the program pdb2pqr with its default options and the PARSE force field [57,58]. Subsequently, a 120×120×120 grid was centered at the system’s center of mass and spread over a 171.36 Å ×171.26 Å ×263.09 Å box, with increments dx = 1.428 Å, dy = 1.42717 Å, and dz = 2.19242 Å, using the VMD program and the PMEPot plugin. Also, for each frame, the chaperonin’s orientation was fitted to the chaperonin’s geometry of the first frame. $\langle \phi_{lr}\rangle$ was finally computed at each grid point $r_{i}$, using the same grid point of each frame, i.e., $\langle \phi_{lr}\left(r_{i}\right)\rangle =\sum_{n}\phi_{lr}\left(t_{n},r_{i}\right)/N$, and the variance was computed as $\sigma^{2}=\langle \phi_{lr}^{2}\left(t_{n},r_{i}\right)\rangle -{\langle \phi_{lr}\left(t_{n},r_{i}\right)\rangle }^{2}$. Both $\langle \phi_{lr}\left(r_{i}\right)\rangle$ and $\sigma^{2}\left(r_{i}\right)$ were rescaled by 0.0258 V/(300 K $k_{B}/e$), since the PMEPot plugin reports data in 300 K $k_{B}/e$ units; here $k_{B}$ is the Boltzmann constant and *e* stands for electron. Further details can be found in Ref. [59].

### 4.3. Control Points

Since we are interested in obtaining meaningful information of $\langle \phi_{lr}\rangle$ inside the chaperonin cavity, we selected specific values of $\langle \phi_{lr}\left(r_{i}\right)\rangle$ from the grid described in Section 4.2. To this end, we sought the largest ellipsoid that fit inside the cavity without touching the interior atoms. To find a single ellipsoid, we applied the following geometric operations sequentially to the first frame out of the 1000 frames described in the previous section. (a) Center the chaperonin at its center of mass. (b) Remove the atoms below the xy plane. (c) Center the remaining chaperonin’s upper half to its center of mass. (d) Rescale coordinates using the size of the box surrounding the upper half (i.e., $r_{\alpha }^{\prime }=\left(x_{\alpha }/A\right)\hat{𝚤}+\left(y_{\alpha }/B\right)\hat{𝚥}+\left(z_{\alpha }/C\right)\hat{k}$, where $r_{\alpha }$ is the radius vector of the α-th atom, and *A*, *B*, and *C* are the dimensions of the box surrounding the upper half). (e) Invert inside out all atom coordinates—$r_{\alpha }^{\prime \prime }=\left(\left(R/\right|r_{\alpha }^{\prime }\left|-1\right)r_{\alpha }^{\prime }\right)$; here, *R* determines a sphere much larger than the sphere needed to enclose all the upper half atoms—. (f) Determine the radius of the sphere that encloses the most outer atom (considering inverted coordinates). Using the information from the previous steps, we determined the dimensions of an ellipsoid by inverting the transformations applied there. To find the largest ellipsoid, in step (c), we added a small translation along the *z* axis, from Δz=−6 Å to Δz=6 Å. The largest ellipsoid was found at Δz=2 Å, i.e., the center of the largest ellipsoid is located 2 Å above the center of mass of the chaperonin’s upper half. Finally, we subtracted 1 Å from each axis of the previous ellipsoid. Returning to the original space, the procedure described above renders an ellipsoid located within the upper cavity of the chaperonin, which touches no single atom. Subsequently, we filtered the grid points of $\langle \phi_{lr}\left(r_{i}\right)\rangle$; we kept only the grid points that met two restrictions: (i) each point $r_{i}$ is located within the ellipsoid and (ii) $\sigma (r_{i})<0.5$ V. A control point $r_{i}=x_{i}\hat{𝚤}+y_{i}\hat{𝚥}+z_{i}\hat{k}$ is inside the ellipsoid when it mets the following condition:

(6) $$\frac{{\left(x_{i}-c_{x}\right)}^{2}}{a^{2}}+\frac{{\left(y_{i}-c_{y}\right)}^{2}}{b^{2}}+\frac{{\left(z_{i}-c_{z}\right)}^{2}}{c^{2}}<1.$$

Here, *a*, *b*, and *c* are the ellipsoid radii and $c=c_{x}\hat{𝚤}+c_{y}\hat{𝚥}+c_{z}\hat{k}$ is the ellipsoid center.

### 4.4. Least Squares Method for Finding the Coefficients of $\langle \phi_{lr}\left(r\right)\rangle$ Using Control Points

In this section, for simplicity, we will use a generic scalar function f(r) to denote either Equation (1) or Equation (2), and we describe the general procedure to determine the coefficients summarized in Table 1. As a general expression, we used a polynomial expansion of the form:

(7) $$f\left(r\right)=\sum_{\alpha =0}^{M}C_{\alpha }x^{a_{\alpha }}y^{b_{\alpha }}z^{c_{\alpha }}$$

For instance, in Equation (1), M=3, $C_{0}=A_{0}$, $a_{0}=b_{0}=c_{0}=0$, $C_{1}=A_{x}$, $a_{1}=1$, $b_{1}=c_{1}=0$, and so on. To determine the set of coefficients $\left\{C_{\alpha }\right\}$, using the least-square method, we used the control points set (denoted here as $\left\{r_{i}\right\}$) filtered as described in Section 4.3. In total, we found N=15,160 control points (see Section 2.3 for further details). As a first step, we assembled the matrix

(8) $$F=\left(\begin{array}{c} f(r_{1})\\ f(r_{2})\\ \vdots \\ f(r_{N}) \end{array}\right),$$

where $f(r_{i})$ is the value obtained from the PMEPot plugin at the control point $r_{i}$, which is inside the ellipsoid found as described in Section 4.3. Since we assume that Equation (7) is valid for all $r_{i}$, then the following equation must be met:

(9) $$F=\left(\begin{array}{c} \sum_{\alpha =0}^{M}C_{\alpha }x_{1}^{a_{\alpha }}y_{1}^{b_{\alpha }}z_{1}^{c_{\alpha }}\\ \sum_{\alpha =0}^{M}C_{\alpha }x_{2}^{a_{\alpha }}y_{2}^{b_{\alpha }}z_{2}^{c_{\alpha }}\\ \vdots \\ \sum_{\alpha =0}^{M}C_{\alpha }x_{N}^{a_{\alpha }}y_{N}^{b_{\alpha }}z_{N}^{c_{\alpha }} \end{array}\right).$$

On the other hand, Equation (9) can be rewritten as:

(10) F=MA,

where

(11) $$A=\left(\begin{array}{c} A_{0}\\ A_{1}\\ \vdots \\ A_{M} \end{array}\right),$$

and the matrix M (whose dimension is N×(M+1)) is constructed from $\left\{r_{i}\right\}$ as:

(12) $$M=\left(\begin{array}{cccc} 1 & x_{1}^{a_{1}}y_{1}^{b_{1}}z_{1}^{c_{1}} & \ldots & x_{1}^{a_{M}}y_{1}^{b_{M}}z_{1}^{c_{M}}\\ 1 & x_{2}^{a_{1}}y_{2}^{b_{1}}z_{2}^{c_{1}} & \ldots & x_{2}^{a_{M}}y_{2}^{b_{M}}z_{2}^{c_{M}}\\ \vdots & \vdots & \ddots & \vdots \\ 1 & x_{N}^{a_{1}}y_{N}^{b_{1}}z_{N}^{c_{1}} & \ldots & x_{N}^{a_{M}}y_{N}^{b_{M}}z_{N}^{c_{M}} \end{array}\right).$$

Once the matrices F and M are assembled, then we can determine the matrix A by several methods, which basically implies finding the pseudoinverse of M, denoted here as $M^{+}$. After finding $M^{+}$, the coefficient matrix is calculated as:

(13) $$A=M^{+}F.$$

The most common formal methods used to determine $M^{+}$ are the QR decomposition and the singular value decomposition (SVD). However, we simply used the C++ armadillo (v. 15.2.4) library to assemble matrices F and M, and to find the matrix A with the function solve.

The coefficients of determination shown in Table 2 were computed as:

(14) $$R^{2}=1-\frac{S_{\mathrm{res}}}{S_{\mathrm{tot}}}=1-\frac{\sum_{i}{\left(Y_{i}-f_{i}\right)}^{2}}{\sum_{i}{\left(Y_{i}-\bar{Y}\right)}^{2}}.$$

Here, $S_{\mathrm{res}}$ is known as the sum of squares of residuals, and _tot_ is the total sum of squares. In addition, in Equation (14), $f_{i}\equiv f\left(r_{i}\right)$ and $\left\{Y_{i}\right\}$ is the set of known values (i.e., the values found via the PMEPot plugin).

## 5. Conclusions

We have performed Molecular Dynamics Simulations of the HSP60/HSP10 chaperonin. The simulation provided information on the time evolution of the system, from which we have measured the long-range electrostatic potential (under the Ewald particle mesh approximation). From this, we have found that throughout an important region of the chaperonin cavity, and as a first approximation, there exists an unidirectional electric field ($(E/\left(V\;\cdot \;{Å}^{-1}\right)\approx -0.0054\hat{𝚤}+0.010\hat{𝚥}-0.162\hat{k}$) that can be considered invariant in time. We have tested the effect of an applied external electric field on a heated protein by performing MD simulations. The heated protein undergoes large deformations, ultimately reaching an unfolded state, compared with the evolution of the same system in the absence of an electric field. Here, the external electric field was extrapolated to the complete space occupied by the protein, including the unfolded geometry. The implications of the existence of an external force that can move entire portions of the proteins are discussed, as well as its possible implications for the protein folding problem.

## Figures and Tables

**Table 1:** Coefficient of each term of Equations (1) and (2), which are valid for all points inside the ellipsoid centered at c/Å$=188.875\hat{𝚤}+188.341\hat{𝚥}+235.258\hat{k}$, and radii a=28.11 Å, b=28.16 Å, and c=22.32 Å.

Coefficient	Value
$A_{0}$	−4.0521 × 10^1^ V
$A_{x}$	5.4487 × 10^−3^ V/Å
$A_{y}$	−1.0490 × 10^−2^ V/Å
$A_{z}$	1.6188 × 10^−1^ V/Å
$A_{0}^{\prime }$	−5.8778 × 10^1^ V
$A_{x}^{\prime }$	4.0177 × 10^−1^ V/Å
$A_{y}^{\prime }$	5.2559 × 10^−2^ V/Å
$A_{z}^{\prime }$	−4.9239 × 10^−2^ V/Å
$A_{xx}^{\prime }$	−7.7542 × 10^−4^ V/Å^2^
$A_{xy}^{\prime }$	2.3151 × 10^−4^ V/Å^2^
$A_{xz}^{\prime }$	−6.2183 × 10^−4^ V/Å^2^
$A_{yy}^{\prime }$	−6.0596 × 10^−4^ V/Å^2^
$A_{yz}^{\prime }$	5.1593 × 10^−4^ V/Å^2^
$A_{zz}^{\prime }$	4.8898 × 10^−4^ V/Å^2^

**Table 2:** Determination coefficients, $R^{2}$, for the fittings of $\langle \phi_{lr}\left(r\right)\rangle$ (Equation (1)) and of $\langle \phi_{lr}^{\prime }\left(r\right)\rangle$ (Equation (2)).

Fitted Potential	$R^{2}$
$\langle \phi_{lr}\left(r\right)\rangle$ (Equation (1))	0.9740
$\langle \phi_{lr}^{\prime }\left(r\right)\rangle$ (Equation (2))	0.9892

## Data Availability

The data presented in this study are available on request from the corresponding author due to the large size of the files.

## Supplementary Materials

The following supporting information can be downloaded at: https://www.mdpi.com/article/10.3390/ijms27073297/s1. The compressed file suppmat.zip contains the set of $\langle \phi_{lr}\rangle$ and σ evaluated at the whole grid, and the filtered set of control points, including coordinates, $\langle \phi_{lr}\left(r_{i}\right)\rangle$ and $\sigma (r_{i})$.

## References

[1] Anfinsen C.B. (1973). Principles that govern the folding of protein chains. Science.

[2] Jumper J., Evans R., Pritzel A., Green T., Figurnov M., Ronneberger O., Tunyasuvunakool K., Bates R., Žídek A., Potapenko A. (2021). Highly accurate protein structure prediction with AlphaFold. Nature.

[3] Baek M., DiMaio F., Anishchenko I., Dauparas J., Ovchinnikov S., Lee G.R., Wang J., Cong Q., Kinch L.N., Schaeffer R.D. (2021). Accurate prediction of protein structures and interactions using a three-track neural network. Science.

[4] Chen S.J., Hassan M., Jernigan R.L., Jia K., Kihara D., Kloczkowski A., Kotelnikov S., Kozakov D., Liang J., Liwo A. (2023). Protein folds vs. protein folding: Differing questions, different challenges. Proc. Natl. Acad. Sci. USA.

[5] Moore P.B., Hendrickson W.A., Henderson R., Brunger A.T. (2022). The protein-folding problem: Not yet solved. Science.

[6] Cao A. (2020). The Last Secret of Protein Folding: The Real Relationship Between Long-Range Interactions and Local Structures. Protein J..

[7] Kikuchi T. (2022). Decoding an Amino Acid Sequence to Extract Information on Protein Folding. Molecules.

[8] Adhada S.T., Sarma S.P. (2024). Slow Conformational Exchange between Partially Folded and Near-Native States of Ubiquitin: Evidence for a Multistate Folding Model. Biochemistry.

[9] Škrbić T., Maritan A., Giacometti A., Rose G.D., Banavar J.R. (2021). Building blocks of protein structures: Physics meets biology. Phys. Rev. E.

[10] Agashe V.R., Shastry M., Udgaonkar J.B. (1995). Initial hydrophobic collapse in the folding of barstar. Nature.

[11] Bhatia S., Udgaonkar J.B. (2022). Heterogeneity in Protein Folding and Unfolding Reactions. Chem. Rev..

[12] Englander S.W., Mayne L. (2014). The nature of protein folding pathways. Proc. Natl. Acad. Sci. USA.

[13] Englander S.W., Mayne L. (2017). The case for defined protein folding pathways. Proc. Natl. Acad. Sci. USA.

[14] Rose G.D., Fleming P.J., Banavar J.R., Maritan A. (2006). A backbone-based theory of protein folding. Proc. Natl. Acad. Sci. USA.

[15] Bolen D.W., Rose G.D. (2008). Structure and Energetics of the Hydrogen-Bonded Backbone in Protein Folding. Annu. Rev. Biochem..

[16] Wetlaufer D.B. (1973). Nucleation, Rapid Folding, and Globular Intrachain Regions in Proteins. Proc. Natl. Acad. Sci. USA.

[17] Baldwin R.L. (1995). The nature of protein folding pathways: The classical versus the new view. J. Biomol. NMR.

[18] Dill K.A., Chan H.S. (1997). From Levinthal to pathways to funnels. Nat. Struct. Biol..

[19] Nassar R., Dignon G.L., Razban R.M., Dill K.A. (2021). The protein folding problem: The role of theory. J. Mol. Biol..

[20] Eaton W.A., Wolynes P.G. (2017). Theory, simulations, and experiments show that proteins fold by multiple pathways. Proc. Natl. Acad. Sci. USA.

[21] Levinthal C. (1968). Are there pathways for protein folding?. J. Chim. Phys..

[22] Levinthal C. (1969). How to fold graciously. Mossbauer Spectrosc. Biol. Syst..

[23] Bai Y., Sosnick T.R., Mayne L., Englander S.W. (1995). Protein Folding Intermediates: Native-State Hydrogen Exchange. Science.

[24] Bai Y., Englander S.W. (1996). Future directions in folding: The multi-state nature of protein structure. Proteins Struct. Funct. Bioinform..

[25] Panchenko A.R., Luthey-Schulten Z., Cole R., Wolynes P.G. (1997). The foldon universe: A survey of structural similarity and self-recognition of independently folding units 11Edited by F. E. Cohen. J. Mol. Biol..

[26] Yang L.Q., Ji X.L., Liu S.Q. (2013). The free energy landscape of protein folding and dynamics: A global view. J. Biomol. Struct. Dyn..

[27] Eaton W.A., noz V.M., Thompson P.A., Chan C.K., Hofrichter J. (1997). Submillisecond kinetics of protein folding. Curr. Opin. Struct. Biol..

[28] Gestaut D., Zhao Y., Park J., Ma B., Leitner A., Collier M., Pintilie G., Roh S.H., Chiu W., Frydman J. (2022). Structural visualization of the tubulin folding pathway directed by human chaperonin TRiC/CCT. Cell.

[29] Hayer-Hartl M., Bracher A., Hartl F.U. (2016). The GroEL-GroES Chaperonin Machine: A Nano-Cage for Protein Folding. Trends Biochem. Sci..

[30] Sadat A., Tiwari S., Sunidhi S., Chaphalkar A., Kochar M., Ali M., Zaidi Z., Sharma A., Verma K., Rao K.B.N. (2022). Conserved and divergent chaperoning effects of Hsp60/10 chaperonins on protein folding landscapes. Proc. Natl. Acad. Sci. USA.

[31] Tang Y.C., Chang H.C., Roeben A., Wischnewski D., Wischnewski N., Kerner M.J., Hartl F.U., Hayer-Hartl M. (2006). Structural Features of the GroEL-GroES Nano-Cage Required for Rapid Folding of Encapsulated Protein. Cell.

[32] Gomez-Llorente Y., Jebara F., Patra M., Malik R., Nisemblat S., Chomsky-Hecht O., Parnas A., Azem A., Hirsch J.A., Ubarretxena-Belandia I. (2020). Structural basis for active single and double ring complexes in human mitochondrial Hsp60-Hsp10 chaperonin. Nat. Commun..

[33] Bie A.S., Cömert C., Körner R., Corydon T.J., Palmfeldt J., Hipp M.S., Hartl F.U., Bross P. (2020). An inventory of interactors of the human HSP60/HSP10 chaperonin in the mitochondrial matrix space. Cell Stress Chaperones.

[34] Vainberg I.E., Lewis S.A., Rommelaere H., Ampe C., Vandekerckhove J., Klein H.L., Cowan N.J. (1998). Prefoldin, a Chaperone that Delivers Unfolded Proteins to Cytosolic Chaperonin. Cell.

[35] Hansen W.J., Cowan N.J., Welch W.J. (1999). Prefoldin-Nascent Chain Complexes in the Folding of Cytoskeletal Proteins. J. Cell Biol..

[36] Humphrey W., Dalke A., Schulten K. (1996). VMD—Visual Molecular Dynamics. J. Mol. Graph..

[37] Ben-Naim A. (2012). Levinthal’s question revisited, and answered. J. Biomol. Struct. Dyn..

[38] Gsponer J., Caflisch A. (2002). Molecular dynamics simulations of protein folding from the transition state. Proc. Natl. Acad. Sci. USA.

[39] Voelz V.A., Singh V.R., Wedemeyer W.J., Lapidus L.J., Pande V.S. (2010). Unfolded-State Dynamics and Structure of Protein L Characterized by Simulation and Experiment. J. Am. Chem. Soc..

[40] Frelih T., Wang B., Plavec J., Šket P. (2020). Pre-folded structures govern folding pathways of human telomeric G-quadruplexes. Nucleic Acids Res..

[41] Durell S.R., Ben-Naim A. (2017). Hydrophobic-hydrophilic forces in protein folding. Biopolymers.

[42] Takagi F., Koga N., Takada S. (2003). How protein thermodynamics and folding mechanisms are altered by the chaperonin cage: Molecular simulations. Proc. Natl. Acad. Sci. USA.

[43] England J.L., Lucent D., Pande V.S. (2008). A Role for Confined Water in Chaperonin Function. J. Am. Chem. Soc..

[44] England J., Lucent D., Pande V. (2008). Rattling the cage: Computational models of chaperonin-mediated protein folding. Curr. Opin. Struct. Biol..

[45] Robustelli P., Piana S., Shaw D.E. (2018). Developing a molecular dynamics force field for both folded and disordered protein states. Proc. Natl. Acad. Sci. USA.

[46] Freddolino L., Park S., Roux B., Schulten K. (2009). Force Field Bias in Protein Folding Simulations. Biophys. J..

[47] Roterman I., Stapor K., Konieczny L. (2024). Model of the external force field for the protein folding process—The role of prefoldin. Front. Chem..

[48] Fischer A.L.M., Tichy A., Kokot J., Hoerschinger V.J., Wild R.F., Riccabona J.R., Loeffler J.R., Waibl F., Quoika P.K., Gschwandtner P. (2024). The Role of Force Fields and Water Models in Protein Folding and Unfolding Dynamics. J. Chem. Theory Comput..

[49] Grosberg A., Attig N., Binder K., Grubmüler H., Kremer K. (2004). Statistical mechamics of protein folding: Some outstanding problems. Computational Soft Matter: From Synthetic Polymers to Proteins.

[50] Berman H.M., Westbrook J., Feng Z., Gilliland G., Bhat T.N., Weissig H., Shindyalov I.N., Bourne P.E. (2000). The Protein Data Bank. Nucleic Acids Res..

[51] Kaminski G.A., Friesner R.A., Tirado-Rives J., Jorgensen W.L. (2001). Evaluation and Reparametrization of the OPLS-AA Force Field for Proteins via Comparison with Accurate Quantum Chemical Calculations on Peptides. J. Phys. Chem. B.

[52] Berendsen H.J.C., Postma J.P.M., van Gunsteren W.F., Hermans J. (1981). Interaction Models for Water in Relation to Protein Hydration. Intermolecular Forces: Proceedings of the Fourteenth Jerusalem Symposium on Quantum Chemistry and Biochemistry Held in Jerusalem, Israel.

[53] (2007). VMD PMEPot Plugin. https://www.ks.uiuc.edu/Research/vmd/plugins/pmepot/.

[54] Aksimentiev A., Schulten K. (2005). Imaging *α*-Hemolysin with Molecular Dynamics: Ionic Conductance, Osmotic Permeability, and the Electrostatic Potential Map. Biophys. J..

[55] Dolinsky T.J., Nielsen J.E., McCammon J.A., Baker N.A. (2004). PDB2PQR: An automated pipeline for the setup of Poisson-Boltzmann electrostatics calculations. Nucleic Acids Res..

[56] Dolinsky T.J., Czodrowski P., Li H., Nielsen J.E., Jensen J.H., Klebe G., Baker N.A. (2007). PDB2PQR: Expanding and upgrading automated preparation of biomolecular structures for molecular simulations. Nucleic Acids Res..

[57] Sitkoff D., Sharp K.A., Honig B. (1994). Accurate Calculation of Hydration Free Energies Using Macroscopic Solvent Models. J. Phys. Chem..

[58] Tang C.L., Alexov E., Pyle A.M., Honig B. (2007). Calculation of pKas in RNA: On the Structural Origins and Functional Roles of Protonated Nucleotides. J. Mol. Biol..

[59] Peña-Ortiz L. (2022). Caracterización del Potencial Electrostático de Largo Alcance en el Interior de la Chaperonina HSP60-HSP10 y su Posible Efecto Sobre el Plegamiento de Proteínas. Master’s Thesis.

